# When the Child Is Not Heard: Children's Rights and Epistemic Injustice in Paediatric Nursing Practice

**DOI:** 10.1111/nup.70106

**Published:** 2026-08-02

**Authors:** Vasiliki Georgousopoulou, Georgios Manomenidis

**Affiliations:** ^1^ Department of Nursing Democritus University of Thrace Alexandroupolis Greece

**Keywords:** child‐centred care, children's rights, dignity, epistemic injustice, nursing ethics

## Abstract

Children's dignity in hospital care is often framed as a matter of privacy, etiquette, communication, or procedural compliance. A more demanding account is needed. Drawing on the philosophical framework of epistemic injustice, this conceptual article argues that dignity‐related harms in paediatric nursing also arise when children are denied credibility, interpretive support, or meaningful authority over knowledge of their own embodied experiences. Children may not always describe pain, shame, embarrassment, fear, or boundary violation through adult‐like verbal testimony. Their experiential knowledge may instead appear through silence, withdrawal, humour, bodily resistance, hesitation, gaze avoidance, or apparently compliant behaviour. When such expressions are dismissed as immaturity, difficulty, exaggeration, or non‐compliance, the child is harmed not only as a vulnerable patient but also as a knower. This reframing connects paediatric dignity with children's rights to participation, privacy, and respect under the United Nations Convention on the Rights of the Child and the EACH Charter. It also positions paediatric nurses as central agents of epistemic repair because nursing work occurs at the intersection of intimate bodily care, sustained relational proximity, communication, documentation, and advocacy. The article proposes an epistemic justice practice bundle for paediatric nursing: credibility‐oriented listening, embodied attunement, developmentally attuned communication, protected communicative spaces, minimal substitution by adult proxies, and voice‐preserving documentation. The analysis extends nursing philosophy by showing that dignity is not only protected around the child but co‐produced through relational practices that make children's experiences visible, credible, and actionable within clinical care.

## 1 Introduction

In paediatric healthcare, privacy and dignity are often treated as matters of etiquette, institutional policy, environmental design, or technical compliance. Curtains, gowns, closed doors, information leaflets, and consent procedures are important safeguards. Yet dignity can still be damaged in ordinary clinical routines: a child left partly undressed during assessment, a procedure explained only to adults, a bodily function discussed at the bedside, multiple staff entering without warning, or a joke intended to reduce tension but experienced by the child as humiliating. Such moments may appear minor from an adult or institutional perspective, but they can be morally and clinically significant for children whose bodies, voices, and choices are already constrained by illness, dependency, and hospital routines.

A children's rights lens clarifies the stakes. Article 12 of the United Nations Convention on the Rights of the Child affirms the child's right to express views freely in all matters affecting them and to have those views given due weight according to age and maturity; Article 16 protects children against arbitrary or unlawful interference with privacy and against attacks on honour and reputation (United Nations 1989). The European Association for Children in Hospital Charter similarly states that children should be treated with tact and understanding, that their privacy should be respected at all times, and that protection should include freedom from physical exposure and treatment that diminishes self‐respect or makes the child feel ridiculous or humiliated (European Association for Children in Hospital 2016). These commitments are not abstract legal ideals. They set normative expectations for everyday paediatric nursing practice.

Recent evidence suggests, however, that children's rights in healthcare remain difficult to implement consistently. A systematic review of healthcare professionals' understanding of children's rights found barriers such as knowledge gaps, misconceptions about consent, resource constraints, heavy workload, adult perceptions of children's capacity, parental dominance, and insufficient training; the same review identified communication, trust‐building, and children's involvement in decisions as key facilitators (Alshammari et al. 2025). In Greece, a study of healthcare professionals in paediatric hospitals found limited awareness of the United Nations Convention on the Rights of the Child and weaknesses in procedures related to gender, privacy, information, infrastructure, complaint recording, and avoidable hospitalisations (Georgousopoulou et al. 2023). These findings indicate that rights‐based care depends on more than formal endorsement. It requires clinical cultures in which children can be heard, believed, and supported to participate (Stålberg et al. 2024).

The central claim developed here is that dignity‐related harms in paediatric nursing are not only ethical, relational, or procedural failures. They can also be epistemic failures. They occur when children are not recognised as credible knowers of their own embodied experiences, or when clinical environments do not provide the interpretive resources that allow children to make sense of and express pain, embarrassment, fear, shame, exposure, and bodily vulnerability. The philosophical framework of epistemic injustice is useful because it shows how people can be wronged specifically in their capacity as knowers when their testimony is unfairly discounted or when shared interpretive resources fail to make their experience intelligible (Fricker 2007; Carel and Kidd 2014). In paediatric care, such wrongs are intensified by age, dependency, developmental assumptions, adult authority, parental mediation, and institutional routines.

Contemporary paediatric dignity scholarship identifies respect, communication, agency or autonomy, and privacy as central components of dignity (Silverstein et al. 2024). Similarly, child‐centred care remains an important but variably defined concept that requires clearer operationalisation across healthcare contexts (Carter et al. 2024). Recent work on children's participation, paediatric teams, and relational autonomy also shows that children are frequently under‐involved in healthcare decisions and that their voices are often mediated through adults (Koller et al. 2024; Hietanen et al. 2025; Matthiesen et al. 2026). The contribution offered here is to theorise these concerns as conditions of knowledge production within nursing care. Paediatric dignity is not only something nurses protect around the child through privacy measures. It is also something nurses co‐produce with the child through listening, interpretation, communication, documentation, and epistemic recognition.

## 2 Children as Epistemic Subjects in Paediatric Care

Children should not be understood only as recipients of care, future adults, or developing subjects whose perspectives must be filtered through professional and parental interpretation. They are also knowers of their own bodily and emotional experiences. Pain, fear, embarrassment, shame, fatigue, exposure, and felt boundary violation are first‐person phenomena. They may be observed by others, but they cannot be fully replaced by parental report or professional judgement. Even when children lack adult linguistic fluency, they remain privileged witnesses to how clinical care is lived from within their own bodies.

Recognising children as epistemic subjects does not require treating them as miniature adults or ignoring developmental differences. It requires developmentally attuned listening. Children's knowledge may be communicated verbally, narratively, affectively, behaviourally, relationally, or bodily. A child may say ‘stop’, turn away, become unusually quiet, laugh nervously, cover their body, avoid eye contact, pull away, ask repeated questions, refuse to change clothes, or appear suddenly compliant. These expressions are not peripheral to care. They may be the form through which the child communicates fear, uncertainty, shame, modesty, pain, or a boundary.

This understanding aligns with paediatric participation research. Children report wanting information, opportunities to express views, and involvement in decisions during hospital stays (Foster et al. 2022). Evidence from paediatric oncology further demonstrates that children can articulate their own values and describe morally challenging situations arising during treatment, highlighting the importance of recognising children as active moral and epistemic subjects (Weiner et al. 2024). Observational work also shows that children participate verbally and non‐verbally in healthcare encounters and that their participation is shaped by the actions of children, parents, and healthcare professionals (Quaye et al. 2019). A systematic review from the child's perspective similarly emphasises that participation depends on space, opportunity, relational support, and recognition of children as actors in healthcare situations (Hietanen et al. 2025). These findings support a shift from asking only whether a child has adult‐like decisional capacity to asking whether the clinical environment has made the child's experiential knowledge expressible and usable.

Nursing philosophy gives this claim additional depth. Carper's account of empirical, ethical, personal, and aesthetic knowing challenged narrow views of nursing knowledge and remains foundational for understanding nursing as more than technical task performance (Carper 1978). This argument is also consistent with later developments in nursing epistemology, which have re‐examined Carper's patterns of knowing and emphasised the continuing importance of personal, aesthetic, relational, and embodied forms of nursing knowledge (Archibald 2012; Thorne 2020). Contemporary work continues to emphasise that nurses draw on multiple patterns of knowing, including experiential, moral, embodied, and relational knowledge (Rafii et al. 2021). In paediatric nursing, such epistemological plurality is especially important because children's distress may not appear as explicit testimony. Nurses may come to know the child through repeated encounters, relational proximity, touch, timing, tone, body language, play, and what remains unsaid. Relational and embodied knowing are therefore not optional additions to paediatric nursing; they are essential ways of apprehending the child's dignity‐relevant experience (Wright and Brajtman 2011).

Epistemic injustice in paediatric nursing is therefore not merely a philosophical problem imported into clinical practice. It is a failure within nursing knowledge itself. When children's embodied expressions are ignored, mislabelled, or overwritten by adult interpretation, nursing loses access to patient knowledge that is morally and clinically important. Protecting dignity requires epistemic practices that preserve the child's first‐person perspective as meaningful evidence within care.

## 3 Embodied Dignity, Privacy, and Institutional Power

Children's dignity in healthcare cannot be understood solely through procedural ethics, information privacy, or institutional rights frameworks because dignity is fundamentally embodied. The body is not only a biological object available for examination and intervention. It is the lived body through which the child experiences vulnerability, exposure, agency, shame, modesty, pain, and recognition (Carel 2016; Fernandez 2020). Illness and hospitalisation can transform a familiar body into a body observed, measured, handled, discussed, and institutionally managed. For children, this transformation is often intensified because adults largely control the timing, location, purpose, and manner of bodily exposure and contact.

Privacy should therefore not be reduced to covering the body or closing a curtain. These actions are necessary, but they are not sufficient. Privacy is also relational and interpretive. It concerns whether the child is approached as an embodied subject with preferences, boundaries, feelings, and developing agency, or as an object of clinical access. A child may be physically covered yet still experience humiliation if a procedure is discussed over them, if staff enter without explanation, if embarrassment is mocked, or if the child's reluctance is treated as an inconvenience. Conversely, even invasive care can be experienced as more dignified when the child receives explanation, preparation, permission to ask questions, opportunities to pause, and reassurance that their bodily boundaries matter.

Institutional power shapes these experiences. In hospital settings, children occupy structurally dependent positions. They are often required to adapt to ward routines, professional schedules, diagnostic priorities, parental decisions, and adult‐defined communication norms. Their participation may depend on whether adults invite them to speak, slow down enough to listen, interpret non‐verbal signals, or allow questions and refusals. Literature on children's participation rights shows that paediatric teams need more practical clarity about how rights should be enacted in everyday care (Koller et al. 2024). Relational autonomy scholarship also shows that children's autonomy in healthcare is socially embedded and that children's and nurses’ perspectives have been underrepresented in the literature (Matthiesen et al. 2026).

The institutional ideal of the quiet, compliant, or ‘good’ child is especially problematic. A child who remains still, stops asking questions, or submits to a procedure may appear cooperative. Yet compliance does not necessarily indicate understanding, trust, assent, or dignity. It may reflect fear, dependency, resignation, previous experiences of being ignored, or uncertainty about whether disagreement is allowed. The ‘good child’ can therefore conceal epistemic vulnerability. Silence and behavioural cooperation should be interpreted cautiously, particularly during intimate or exposing care. For paediatric nurses, the ethical question is not simply whether the child resisted, but whether the child was given a meaningful opportunity to understand, ask, pause, object, express bodily discomfort, and have that expression taken seriously.

## 4 Epistemic Injustice as a Framework for Paediatric Dignity

### 4.1 Testimonial Injustice and Age‐Based Credibility Deficits

Testimonial injustice occurs when a speaker receives less credibility than they deserve because of prejudice or identity‐based assumptions (Fricker 2007). In paediatric healthcare, credibility deficits may be produced by assumptions that children are immature, dramatic, confused, suggestible, attention‐seeking, or incapable of reliably describing their experiences. Such assumptions are not always explicit. They may appear in subtle clinical habits: asking the parent first and the child only afterwards, translating the child's words into adult categories without checking meaning, treating crying as behavioural difficulty, reading embarrassment as shyness, or documenting distress as non‐compliance.

The privacy and dignity domain is particularly vulnerable to testimonial injustice because embarrassment often appears clinically secondary. A child who says ‘I do not want that’, ‘I hate this’, or ‘please do not look’ may be treated as obstructing care rather than communicating a boundary. A child who covers the body, turns away, laughs nervously, or becomes silent may be assumed to be shy rather than humiliated. In these moments, the child is not only emotionally distressed. The child is denied appropriate uptake as a knower of their own embodied experience. The harm is intensified when adult accounts become the default authoritative narrative and the child's first‐person testimony is treated as optional, unreliable, or merely behavioural.

Healthcare applications of epistemic injustice have shown that patients can be discredited when illness, vulnerability, emotion, or stigma are assumed to undermine reliability (Carel and Kidd 2014). In nursing and mental health contexts, epistemic injustice has also been used to analyse silencing, doubting, and dismissing service users’ knowledge (Fisher 2024; Haghiri‐Vijeh 2025). Paediatric care adds a distinctive dimension: age and dependency can make credibility deficits appear benevolent or protective. Yet protection becomes ethically unstable when it authorises adults to disregard the child's account precisely in matters where the child is the primary knower.

### 4.2 Hermeneutical Injustice and the Under‐Description of Bodily Vulnerability

Hermeneutical injustice arises when a person is disadvantaged because shared interpretive resources are insufficient for making sense of an experience (Fricker 2007). Children may be especially vulnerable to this form of injustice because they may lack vocabulary for humiliation, bodily boundaries, modesty, fear, shame, or loss of control. They may also be situated in clinical environments that do not offer age‐appropriate ways to express these experiences. A child who cannot say ‘I feel humiliated because my body is exposed’ may say ‘no’, ‘stop’, ‘I am cold’, ‘I want my mum’, or nothing at all. Without interpretive support, the dignity‐relevant meaning of these expressions may remain invisible.

Hermeneutical injustice is not simply a problem inside the child's mind. It is produced by clinical environments that privilege adult language, biomedical categories, speed, and procedural completion. If the ward offers no child‐friendly explanations, no visual or play‐based communication supports, no permission to pause, no private space away from adult pressure, and no routine invitation to describe bodily discomfort, then children may lack the practical conditions needed to interpret and communicate their experiences. Child‐centred communication research supports the importance of developmentally appropriate strategies, including playful communication, simplified explanations, and relational engagement (Osei Appiah et al. 2022; DeCosta et al. 2022; Sillero Sillero et al. 2024). These are not merely communication techniques. They are epistemic supports that help children make their experiences clinically intelligible.

Recent conceptual caution is also necessary. Scholars have argued that healthcare applications of epistemic injustice should avoid overextension and should distinguish carefully between philosophical claims, empirical evidence, and clinical interpretation (Nielsen et al. 2025). This caution strengthens rather than weakens the present argument. Not every failure of communication is automatically epistemic injustice. The concept is most useful when children's credibility is unfairly downgraded because of age‐based assumptions, or when clinical systems fail to provide interpretive resources that children need to express experiences that are morally and clinically significant. Used with such precision, epistemic injustice offers a valuable lens for paediatric nursing ethics.

### 4.3 Beyond Fricker: Expanding Epistemic Injustice for Paediatric Nursing

Although Fricker's distinction between testimonial and hermeneutical injustice remains the foundational philosophical framework for understanding epistemic wrongs in healthcare, subsequent scholarship has demonstrated that epistemic injustice extends beyond interpersonal credibility deficits and limitations in shared interpretive resources. Contemporary philosophical and nursing scholarship increasingly recognises that epistemic exclusion is often embedded within broader institutional, professional, and sociopolitical structures that determine whose knowledge is regarded as credible, whose experiences become intelligible, and whose perspectives are systematically marginalised. Rather than resulting solely from individual prejudice, epistemic injustice may be reproduced through organisational cultures, professional norms, and everyday clinical routines that shape whose voices are heard and whose experiences are discounted.

This broader understanding is particularly evident within contemporary nursing philosophy. Recent work published in *Nursing Philosophy* has similarly shown that nursing knowledge is shaped by what is heard, silenced, recognised, or excluded within professional and institutional cultures (Dillard‐Wright et al. 2023; Haghiri‐Vijeh 2025). Dillard‐Wright et al. (2023) argue that nursing is characterised not only by what it knows but also by what it systematically chooses not to know. They describe practices of epistemic silence and silencing whereby particular experiences remain invisible because dominant professional assumptions determine which forms of knowledge are recognised as legitimate. Likewise, Haghiri‐Vijeh (2025) demonstrates that epistemic injustice is embedded within nurses' encounters with socially marginalised populations, illustrating how discrimination may occur through subtle processes of discrediting patients' lived experiences rather than through overt prejudice alone. Together, these perspectives suggest that epistemic justice requires nurses to critically examine not only interpersonal communication but also the institutional cultures, organisational practices, and power relations that shape clinical knowledge. Collectively, these contributions demonstrate that contemporary nursing philosophy increasingly conceptualises epistemic justice as a relational, institutional, and ethical responsibility rather than solely an individual communicative competence.

Paediatric nursing illustrates why this theoretical expansion is necessary. Children's epistemic vulnerability cannot be explained solely through age‐related credibility deficits. Unlike most adult patients, children occupy structurally dependent positions within healthcare systems. Their experiences are routinely interpreted through parents, evaluated according to developmental expectations, filtered through professional judgement, and constrained by institutional routines established by adults. Consequently, children's epistemic marginalisation emerges simultaneously through interpersonal interactions, organisational practices, and broader assumptions regarding childhood, competence, dependence, and authority. Children's experiential knowledge may therefore become marginalised not because children lack meaningful experiences, but because healthcare systems frequently privilege adult interpretations over children's own accounts.

Moreover, children's epistemic vulnerability frequently intersects with broader social inequities. Race, ethnicity, language, disability, migration status, socioeconomic disadvantage, and cultural background may further influence whether children's experiences are regarded as credible and worthy of clinical attention. Flores and Tomany‐Korman (2008), for example, demonstrated persistent racial and ethnic disparities in children's access to healthcare and quality of care, suggesting that not all children occupy the same epistemic position within healthcare systems. An intersectional perspective therefore complements a children's rights approach by recognising that multiple forms of marginalisation may simultaneously influence children's opportunities to participate meaningfully in healthcare decisions and to have their experiences believed.

Building on these developments, this article argues that paediatric nursing requires an expanded understanding of epistemic injustice. While Fricker's concepts of testimonial and hermeneutical injustice remain indispensable for identifying epistemic harms, paediatric care also demands attention to the developmental, relational, institutional, and structural conditions through which children's knowledge is either enabled or constrained. The contribution offered here is therefore not simply the application of Fricker's philosophical framework to paediatric nursing. Rather, it extends contemporary nursing philosophy by conceptualising children as embodied, relational, and developmentally situated knowers whose experiences are co‐produced, recognised, and translated through nursing relationships. Protecting children's dignity therefore requires not only ethical sensitivity but also relational justice, epistemic humility, and sustained critical reflection on how nursing practice, institutional cultures, and healthcare systems shape whose knowledge becomes visible, credible, and actionable within clinical care (Dillard‐Wright et al. 2023; Tembo 2025).

### 4.4 From Epistemic Harms to Children's Rights Harms

Epistemic injustice provides a bridge between ordinary clinical interactions and the realisation of children's rights. Rights to participation, dignity, privacy, information, and assent are not realised only through policies or signatures. They depend on whether the child is recognised as someone whose perspective can matter. Article 12 of the United Nations Convention on the Rights of the Child requires not only that children be allowed to express views, but that those views be given due weight (United Nations 1989). Lundy's influential model of participation similarly emphasises that voice alone is insufficient without space, audience, and influence (Lundy 2007). In paediatric nursing terms, the child must have communicative space, a listening audience, and some possibility that their expressed experience will shape care.

Epistemic harms become rights harms when children are formally present but substantively unheard. A child may be in the room during a discussion yet excluded from the explanation. A child may be asked whether they are ‘okay’ in a rushed tone that makes dissent unlikely. A child may assent procedurally while lacking a safe opportunity to express fear or uncertainty. A child may be documented as ‘cooperative’ even though cooperation masks embarrassment or resignation. These situations can satisfy the appearance of inclusion while failing to protect the child's dignity as a participant in care.

The connection between epistemic justice and rights is especially important for assent. Assent is ethically meaningful only when children receive developmentally appropriate information, have opportunities to ask questions, and can express disagreement or discomfort without fear that they will be dismissed. Contemporary scholarship on paediatric assent continues to highlight the importance of transparent, developmentally appropriate communication and documentation (Takahashi and Menikoff 2026). In clinical practice, assent should therefore be understood not as a single verbal expression but as a relational process that includes preparation, interpretation of verbal and non‐verbal cues, permission to pause, and careful attention to dissent.

Dignity, privacy, and participation should consequently be treated as interdependent. Privacy without voice can become bodily management. Participation without privacy can expose the child to pressure or embarrassment. Dignity without epistemic recognition can remain a professional ideal rather than a lived experience for the child. Rights‐based paediatric nursing requires clinical practices that enable children to be heard, understood, believed, and responded to in ways appropriate to their age, maturity, context, and communicative style.

While childhood itself creates conditions of epistemic vulnerability, children's opportunities to be heard, believed, and meaningfully involved in healthcare are not shaped by age alone. Contemporary scholarship increasingly recognises that children's experiences are influenced by the intersection of multiple social identities and structural inequalities, including race, ethnicity, migration status, citizenship, socioeconomic disadvantage, disability, language, gender, and sexuality. These intersecting characteristics may compound children's vulnerability to epistemic injustice by further reducing the credibility attributed to their experiences or limiting their opportunities to participate in healthcare encounters (Haghiri‐Vijeh 2025; Tembo 2025).

An intersectional perspective is particularly important because healthcare systems do not afford all children equal opportunities to exercise their rights or participate meaningfully in care. Evidence consistently demonstrates that racial and ethnic minority children experience disparities in healthcare access, communication, quality of care, and clinical outcomes (Flores and Tomany‐Korman 2008). In particular, evidence from the United States indicates that Black children experience disparities in pain assessment, symptom management, and clinical decision‐making, illustrating how racialised assumptions may influence whose suffering is recognised and whose testimony is afforded credibility. Although these inequities have primarily been examined within the broader literature on health disparities, they may also be understood as manifestations of epistemic injustice, whereby children's experiential knowledge is differentially valued according to socially constructed identities.

Similarly, migrant and refugee children may encounter additional epistemic barriers because language differences, cultural misunderstandings, uncertain citizenship status, and reliance on adult interpreters can restrict opportunities to communicate directly with healthcare professionals. Children with disabilities or neurodevelopmental conditions may likewise experience credibility deficits when expressions of pain, distress, or discomfort are attributed primarily to their diagnosis rather than recognised as meaningful testimony. Gender identity and sexual orientation may further shape adolescents’ healthcare experiences when their concerns are misunderstood, dismissed, or interpreted through normative assumptions about childhood, family life, and identity (Haghiri‐Vijeh 2025).

Recognising these intersecting forms of marginalisation does not diminish the universality of children's rights; rather, it reinforces a rights‐based approach by acknowledging that equal rights do not necessarily translate into equal opportunities to exercise those rights. Consequently, protecting children's dignity requires more than equal treatment; it requires equity‐oriented paediatric nursing that recognises that some children need additional communicative, relational, and institutional support before they can participate as credible epistemic agents in clinical decision‐making. Accordingly, epistemic justice in paediatric nursing should be understood not only as an interpersonal ethical obligation but also as a structural commitment to equity, requiring culturally responsive, anti‐discriminatory, and institutionally accountable practices that actively identify and address the social conditions limiting particular groups of children from exercising their rights to participation, information, privacy, and respect (Dillard‐Wright et al. 2023; Flores and Tomany‐Korman 2008; Haghiri‐Vijeh 2025; Slemon et al. 2025; Tembo 2025; Woods 2012).

## 5 Paediatric Nursing as Epistemic Repair

If dignity‐related harms can be epistemic, paediatric nursing can also be a practice of epistemic repair. Epistemic repair refers here to relational and institutional practices that restore credibility, interpretive support, communicative legitimacy, and clinical uptake to children whose experiences risk being ignored, minimised, or translated away. Nurses are especially important because they often spend sustained time with children, provide intimate bodily care, mediate between families and multidisciplinary teams, observe changes over time, and produce documentation that shapes future interpretations of the child.

Nursing work is therefore epistemically pivotal. During bathing, toileting assistance, dressing changes, catheterisation, physical examination, perioperative preparation, medication administration, or symptom assessment, nurses encounter children in moments of bodily vulnerability. These are not merely technical encounters. They are moments in which the child's dignity can be protected, damaged, or repaired. The following practice bundle translates the conceptual analysis into nursing actions. Table 1.

**Table 1:** Epistemic justice practice bundle for paediatric nursing.

Practice domain	Epistemic risk addressed	Nursing actions
Credibility‐oriented listening	Children's words or cues are dismissed as immaturity, exaggeration, shyness, or non‐compliance.	Ask the child directly where possible; treat silence, hesitation, refusal, humour, or withdrawal as potentially meaningful; check interpretations before labelling behaviour.
Embodied attunement	Bodily distress is seen as procedural inconvenience rather than communication.	Attend to posture, gaze, withdrawal, body covering, tension, and sudden compliance; pause when feasible; explain touch and exposure before they occur.
Developmentally attuned communication	Adult language and clinical pace make children's experiences difficult to express.	Use age‐appropriate language, play, drawings, stories, visual tools, pain or emotion scales, concrete choices, and repeated checks of understanding.
Protected communicative spaces	Adult or parental accounts automatically replace the child's account.	Create opportunities for the child to speak directly; minimise proxy substitution for first‐person experiences such as pain, fear, embarrassment, and bodily boundaries.
Voice‐preserving documentation	Clinical records transmit adult behavioural labels and erase the child's perspective.	Document the child's own words where possible; contextualise distress; record preferences, boundaries, and what helped preserve dignity.
Institutional support	Epistemically just care is undermined by workload, space, culture, or lack of training.	Embed children's rights, assent, privacy, communication tools, and epistemic justice in policies, staffing, ward design, education, and audit standards.

### 5.1 Credibility‐Oriented Listening

Credibility‐oriented listening means approaching the child's account as potentially meaningful before translating it into adult categories. It requires resisting premature labels such as ‘difficult’, ‘dramatic’, ‘attention‐seeking’, or ‘non‐compliant’. Such labels may sometimes describe behaviour, but they can also conceal fear, shame, pain, misunderstanding, or a boundary violation. Credibility‐oriented listening begins with the assumption that the child's words, silence, posture, hesitation, or refusal may be evidence of something important. The nurse's task is to investigate meaning rather than to secure compliance as quickly as possible.

This does not mean that every child's request can be granted or that clinical necessity disappears. Rather, it means that even when care must proceed, the child's experience remains epistemically relevant. A child who refuses a dressing change may still need the dressing changed, but the refusal may reveal pain, fear, embarrassment, mistrust, lack of explanation, or a previous traumatic experience. Credibility‐oriented listening allows clinical necessity and epistemic respect to coexist.

### 5.2 Embodied Attunement and Relational Presence

Embodied attunement involves sensitivity to the ways children communicate through the body. Nurses may notice that a child becomes still, turns away, clenches hands, avoids eye contact, laughs nervously, pulls clothing tightly, holds a parent's hand, or becomes unusually compliant. Such cues should not be reduced to behaviour management. They may be forms of embodied testimony. Relational presence requires nurses to slow the encounter where possible, explain what will happen, check the child's understanding, notice discomfort, and respond in ways that preserve the child's sense of being recognised.

Embodied attunement is particularly important during procedures involving exposure or touch. A dignity‐sensitive approach includes preparing the child before exposure, explaining who needs to be present and why, limiting unnecessary observers, asking permission before touching where feasible, covering body parts not involved in care, naming modesty as legitimate, and allowing the child to choose among clinically safe options. These practices communicate that the child's body is not merely available to the institution.

### 5.3 Developmentally Attuned Communication

Developmentally attuned communication creates the conditions in which children's experiences can become expressible. It may include short explanations, visual supports, dolls, drawings, stories, play‐based communication, pain scales, emotion cards, repeated checks of understanding, and concrete choices. It also requires explicit permission to speak: ‘Tell me if you want me to stop for a moment’; ‘You can say if you feel embarrassed’; ‘Would you like me to explain this to you first?’; ‘Who would you like in the room?’; ‘Is there a part of this that worries you?’ These invitations help children understand that their experience is relevant to care.

Communication should also avoid false reassurance and coercive friendliness. Phrases such as ‘be brave’, ‘good children do not cry’, or ‘this will not hurt’ can undermine epistemic trust if the child's actual experience differs. More respectful formulations acknowledge difficulty without shaming: ‘This may feel uncomfortable’; ‘You can tell me what you feel’; ‘We need to do this, but we can make a plan together’. Developmentally attuned communication is therefore not simply a method for gaining cooperation. It is a rights‐based and epistemically just practice.

### 5.4 Protected Communicative Spaces and Minimal Substitution

Children's voices are often mediated by parents and professionals. Parental knowledge is important and may be indispensable, especially for younger children, children with communication difficulties, or children who rely on parents for emotional security. However, parental substitution should be minimal rather than automatic. Where developmentally and clinically appropriate, nurses should create opportunities for the child to speak directly, including brief protected moments in which the child can express embarrassment, fear, preferences, or questions without immediate adult correction or interpretation.

Protected communicative spaces do not require excluding parents inappropriately. They require careful negotiation of who speaks, who answers first, and whose interpretation is recorded. A practical rule is to seek the child's account whenever the issue concerns first‐person experience: pain, fear, embarrassment, fatigue, bodily exposure, touch, preferences, and perceived boundaries. Adult proxy accounts can supplement but should not routinely replace the child's perspective.

### 5.5 Voice‐Preserving Documentation

Clinical documentation is not epistemically neutral. Records shape how future professionals understand the child, interpret behaviour, and respond to distress. When documentation replaces the child's account with adult shorthand, the child's voice can disappear. Notes such as ‘refused care’, ‘uncooperative’, or ‘difficult’ may be efficient, but they can also transmit credibility deficits across shifts and services. Voice‐preserving documentation records what the child said or communicated, the context in which distress occurred, and what helped.

For example, instead of ‘child uncooperative during wound care’, documentation could state: “Child covered abdomen and said, ‘I do not want everyone looking’. Dressing change paused; non‐essential staff left; procedure explained using simple language; child agreed to continue with parent present and body covered except wound area.” Such documentation preserves the child's experiential knowledge and transforms distress from a behavioural problem into actionable clinical information. It also allows future care to be planned in ways that prevent repeated dignity harms.

### 5.6 Institutional and Educational Responsibilities

Epistemic repair cannot depend only on individual nurses' sensitivity. Institutions must make epistemically just care possible. This includes staffing levels and ward routines that allow time for explanation, physical spaces that protect privacy, policies that limit unnecessary observers during intimate care, communication tools for different ages and abilities, training on children's rights and assent, and documentation standards that preserve children's voices. Rights education should move beyond legal awareness toward practical rehearsal of how to listen, interpret, document, and advocate when children communicate distress indirectly.

Education in paediatric nursing should therefore include epistemic injustice, children's rights, relational autonomy, child‐centred communication, trauma‐informed practice, and embodied dignity. Simulation and reflective learning can help nurses recognise how age‐based assumptions, institutional efficiency, and adult dominance may silence children. Interprofessional education is also needed because children's epistemic marginalisation often occurs across team interactions, not only in one nurse‐child dyad.

Importantly, epistemic injustice should not be understood solely as the consequence of individual communication failures or personal biases. Healthcare organisations themselves may inadvertently reproduce epistemic injustice through institutional structures, policies, and routine practices that privilege efficiency over participation and standardisation over individual experience (Dillard‐Wright et al. 2023; Tembo 2025). High workload, inadequate nurse staffing, time pressures, fragmented continuity of care, documentation systems centred primarily on biomedical outcomes, hierarchical decision‐making, and limited opportunities for children's direct participation may all reduce the likelihood that children's perspectives are actively elicited, documented, and incorporated into clinical decision‐making. As Dillard‐Wright et al. (2023) argue, professional cultures inevitably shape what nurses come to recognise as legitimate knowledge, while organisational priorities may unintentionally silence experiences that do not fit dominant clinical narratives. In such contexts, children's voices are not intentionally ignored but become structurally marginalised because healthcare systems prioritise organisational demands over meaningful engagement with children's lived experiences. This perspective is consistent with contemporary philosophical accounts that conceptualise justice as a relational practice requiring recognition of individuals as legitimate knowers whose experiences deserve ethical and clinical consideration (Tembo 2025). Moreover, Dillard‐Wright et al. (2023) και Tembo (2025) emphasise that epistemic injustice should be understood not merely as an interpersonal communication problem but as a phenomenon that may also emerge from institutional arrangements and healthcare practices that systematically disadvantage particular groups of patients. Consequently, addressing epistemic injustice in paediatric nursing requires organisational commitment to children's rights, adequate staffing, participatory models of care, documentation practices that preserve children's voices, and educational initiatives that encourage nurses to critically reflect on institutional sources of epistemic exclusion (Dillard‐Wright et al. 2023; Haghiri‐Vijeh 2025; Tembo 2025).

## 6 Conceptual Contribution to Nursing Philosophy

The conceptual contribution is threefold. First, paediatric dignity is reframed as an epistemic as well as an ethical and relational phenomenon. Dignity is damaged when children's accounts of pain, shame, fear, exposure, or boundary violation are not believed or are not made intelligible within care. Second, children are positioned as embodied epistemic subjects whose testimony may be verbal, non‐verbal, affective, behavioural, or relational. This challenges adult‐centred assumptions that only articulate, rational, adult‐like speech counts as clinically meaningful testimony. Third, paediatric nursing is positioned as a practice of epistemic repair because nurses can make children's experiences visible, credible, and actionable through listening, embodied attunement, communication, advocacy, and documentation. This reframing also extends nursing epistemology. Nursing knowledge includes more than the collection of observable signs, parental histories, and biomedical data. It includes the interpretive work of understanding what matters to the child in moments of bodily vulnerability. The nurse's ethical role is not only to protect the child from exposure but also to protect the child's capacity to be known on their own terms. In this sense, dignity is co‐produced: it emerges through relationships in which the child's embodied experience is recognised, interpreted with humility, and allowed to influence care.

Furthermore, this framework suggests that paediatric epistemic injustice should be understood as developmentally mediated and intersectionally situated, emerging through the interaction of children's developmental position with broader structural inequities that shape whose knowledge becomes credible within healthcare.

## 7 Limitations and Directions for Future Research

The argument is conceptual and does not claim to demonstrate the prevalence of epistemic injustice in paediatric nursing. Empirical research is needed to examine how children, parents, nurses, and other professionals understand dignity‐related communication, bodily exposure, assent, and documentation in different clinical settings. Observational studies could explore how children's verbal and non‐verbal expressions are taken up during intimate care. Interview and participatory methods could examine children's own accounts of being heard or unheard in the hospital. Documentation audits could assess whether clinical records preserve children's words and preferences or translate them into adult behavioural labels.

Further research should also examine differences across age, disability, neurodivergence, culture, language, gender, chronic illness, intensive care, emergency care, oncology, surgery, and palliative care. Children who communicate without speech, children with developmental disabilities, migrant children, and children from marginalised communities may face additional layers of testimonial and hermeneutical vulnerability. The epistemic justice practice bundle proposed here should therefore be tested, refined, and adapted with children, families, nurses, and multidisciplinary teams.

## 8 Conclusion

Children's dignity in paediatric healthcare cannot be adequately protected through procedural privacy measures, rights policies, or good intentions alone. Dignity also depends on whether children are recognised as knowers of their own embodied experiences. When a child's pain, embarrassment, shame, fear, silence, hesitation, or boundary‐setting is dismissed, misinterpreted, or overwritten by adult accounts, the harm is epistemic as well as ethical. The child is not only exposed or unheard; the child is denied appropriate authority over the meaning of their own experience. Paediatric nurses are central to preventing and repairing such harms. Through intimate care, sustained relational presence, communication, interpretation, documentation, and advocacy, nurses help determine whether children's experiences become visible and actionable within clinical practice. Epistemic justice is therefore not peripheral to paediatric nursing. It is a core condition of dignified, rights‐based, child‐centred care.

## Funding

The authors have nothing to report.

## Ethics Statement

The authors have nothing to report.

## Conflicts of Interest

The authors declare no conflicts of interest.

## Data Availability

The authors have nothing to report.
